# Supplementary material for: Nest Turrets of Acromyrmex Grass-Cutting Ants: Micromorphology Reveals Building Techniques and Construction Dynamics
Source: Insects. 2020 Feb 24;11(2):140. doi: 10.3390/insects11020140 (PMC7073553; doi:10.3390/insects11020140)
Supplement: Supplementary file 1 [file insects-11-00140-s001.zip › Suppl Material.pdf]

SUPPLEMENTARY MATERIAL

Cosarinsky, Römer and Roces. Nest turrets of *Acromyrmex* grass-cutting ants: micromorphology reveals building techniques and construction dynamics

Figure S1

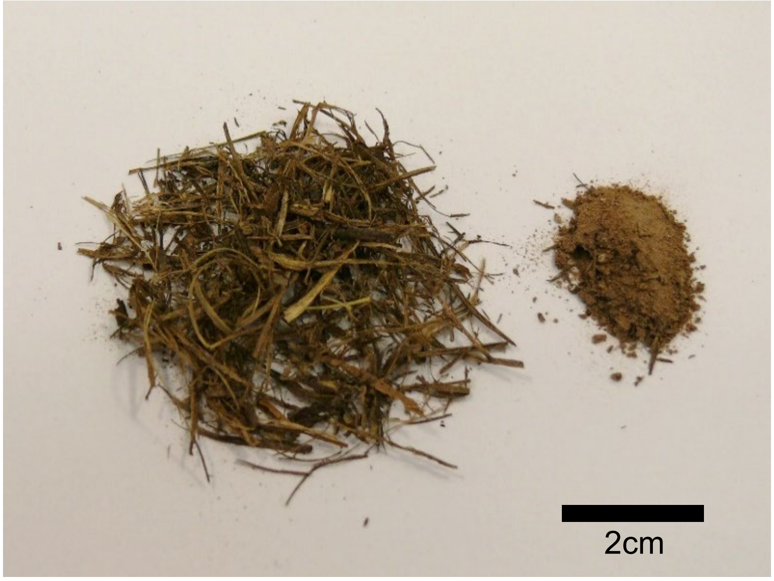

Figure S1: Example of the building materials used for the construction of a single turret. One original turret was first dissolved in demineralized water, and thereafter its components separated into plant (mostly grass blades) and soil materials, and dried overnight at 50°C to quantify their mass.

SUPPLEMENTARY MATERIAL

Figure S2

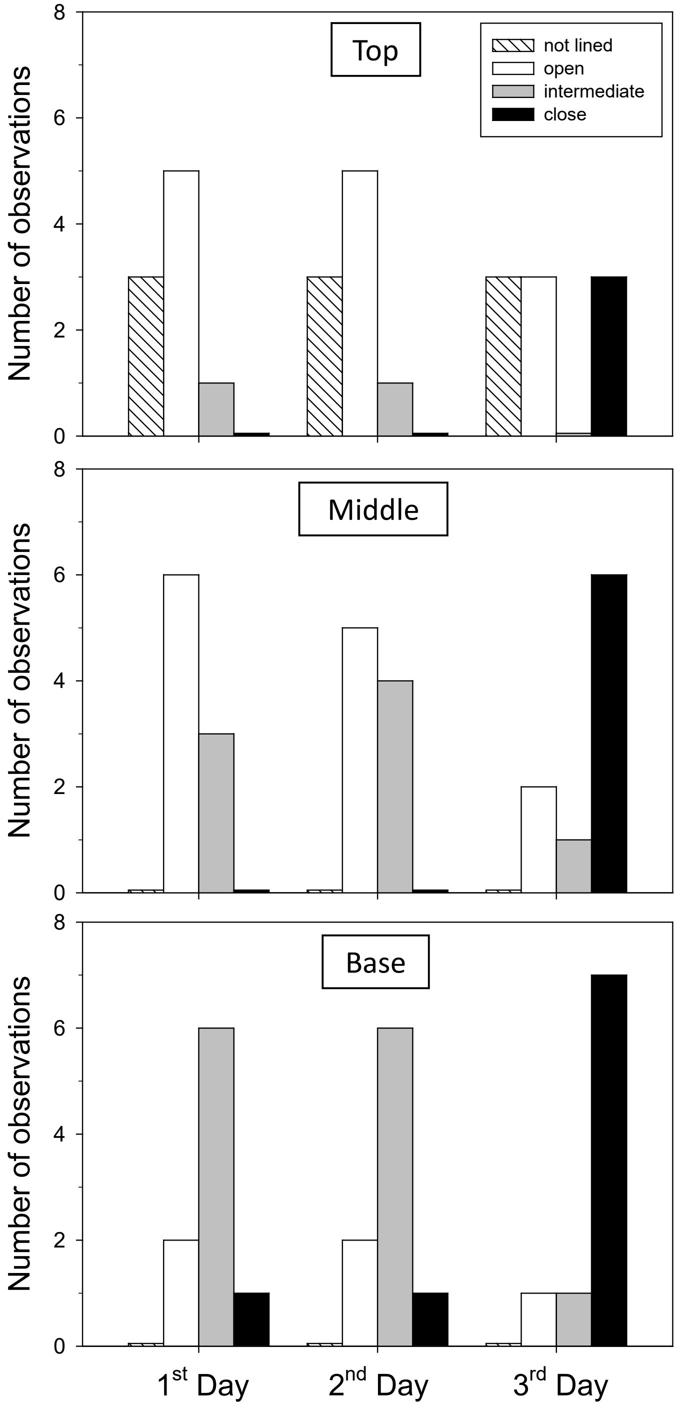

Figure S2: Lining fabrics: shown is the occurrence of the less common lining fabrics observed in the rebuilt turrets over three days, at the top, middle and base of the turret structure (n=9 turrets analyzed). The most common lining fabrics are presented in the main body of the article, in Figure 4.

SUPPLEMENTARY MATERIAL

Figure S3

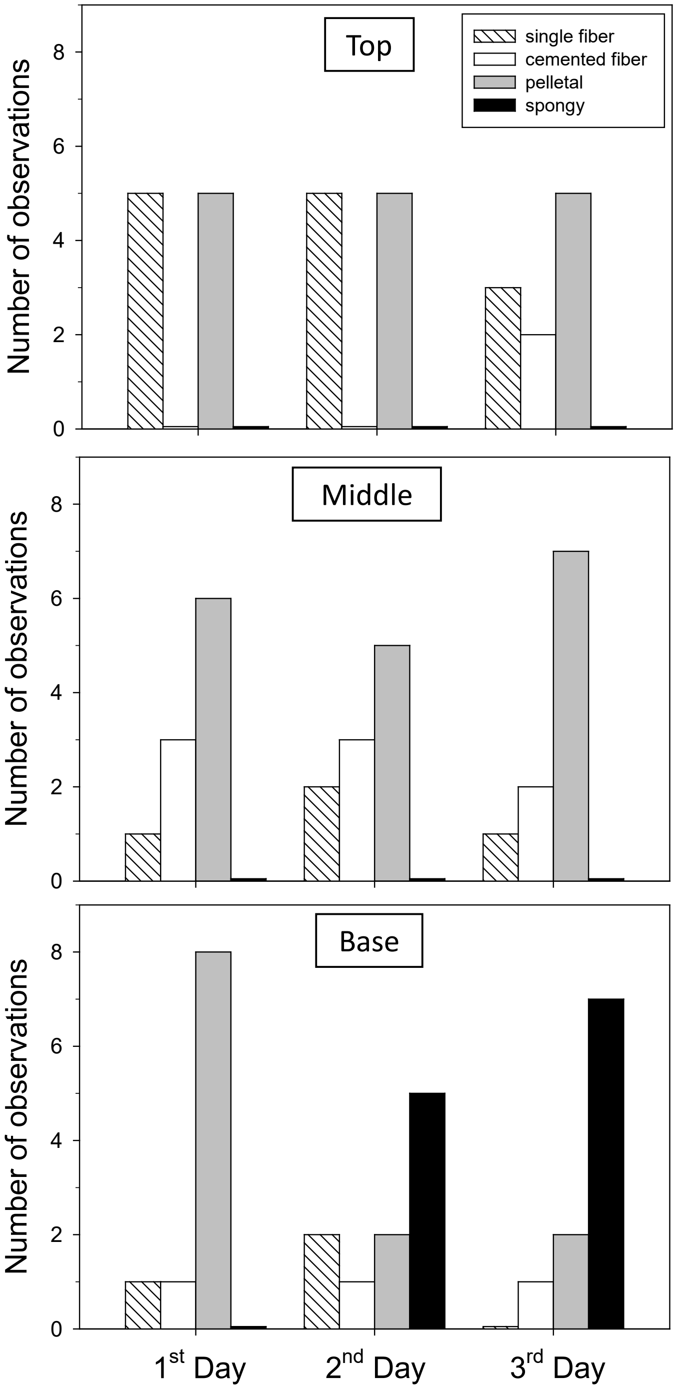

Figure S3: Inner wall microstructures: shown is the occurrence of the less common microstructures observed in the inner walls of the rebuilt turrets over three days, at the top, middle and base of the turret structure (n=10 turrets analyzed). The most common microstructures are presented in the main body of the article, in Figure 5.
